# Stakeholders’ experiences of the implementation of a multifactorial intervention for safe patient handling and movement: a qualitative explorative and descriptive study

**DOI:** 10.1186/s12913-026-16068-8

**Published:** 2026-11-06

**Authors:** Emma Nilsing-Strid, Jan Sandqvist, Glykeria Skamagki, Charlotte Wåhlin

**Affiliations:** 1 Department of Occupational and Environmental Medicine, Faculty of Medicine and Health, Örebro University, Örebro, Sweden; 2 Department of Health, Medicine and Caring Sciences, Linköping University, Norrköping, Sweden; 3 School of Sport, Exercise and Rehabilitation Sciences, College of Life and Environmental Sciences, University of Birmingham, Birmingham, UK; 4 Department of Health, Medicine and Caring Sciences, Linköping University, Linköping, Sweden; 5 Clinical Department of Occupational and Environmental Medicine in Linköping, Region Östergötland, Linköping, Sweden; 6 Unit of Intervention and Implementation Research for Worker Health, Institute for Environmental Medicine, Karolinska Institute, Stockholm, Sweden

**Keywords:** Implementation science, Qualitative research, Moving and lifting patients, Leadership, Ergonomics, Occupational health, Practice guideline

## Abstract

**Background:**

Despite growing evidence for the effectiveness of safe patient handling and movement (PHM) interventions, there is limited insight into implementation and sustainment in routine healthcare. By focusing on stakeholders directly involved in implementation following a cluster randomised controlled trial (RCT), this study provides insight into the mechanisms through which a safe PHM intervention is enacted in practice and how the implementation processes contribute to implementation.

**Aim:**

To explore stakeholders’ experiences of implementing a multifactorial intervention for safe PHM, and to examine the determinants and perceived outcomes influencing its integration and sustained use in clinical practice.

**Methods:**

A qualitative explorative design was employed, using individual interviews with 25 implementation stakeholders (leaders, external facilitators, and implementation team members). The stakeholders were recruited from a cluster RCT evaluating the effectiveness and implementation of a multifactorial safe PHM intervention in Swedish regional and municipal healthcare units. Data were analysed with qualitative deductive-inductive content analysis using the Consolidated Framework for Implementation Research (CFIR) as a theoretical framework.

**Results:**

The analysis resulted in five deductive categories corresponding to CFIR constructs (innovation, inner setting, individuals, implementation process, and actual implementation outcomes) and 13 inductively developed categories. Determinants influencing the implementation of a safe PHM practice were described. The implementation outcomes reflected implementation, enactment of the intervention in practice, and conditions for sustained use, manifested through perceived changes in safety culture, work routines, and use of patient handling equipment.

**Conclusions:**

This study identified key determinants and perceived outcomes influencing the implementation of safe PHM practices. The interdependence between leadership, facilitation, and participants’ active engagement with knowledge shaped the implementation process, enabling translation into practice. Outcomes were reflected in the perceived integration of practices into clinical routines and changes in safety culture, indicating implementation. These findings highlight the importance of organisational and social processes in supporting the sustained use of new practices.

**Trial registration:**

This study is part of a cluster randomised controlled trial, registered at ClinicalTrials.gov on 2 November 2022 (ref NCT05276180).

**Supplementary Information:**

The online version contains supplementary material available at https://doi.org/10.1186/s12913-026-16068-8.

## Background

Musculoskeletal disorders are one of the most prevalent occupational health issues among healthcare workers (HCWs) [1, 2], with patient handling and movement (PHM) being a major contributor to physical workload and injury risk [3–6]. Staff involved in direct patient care are repeatedly exposed to mechanical loads during lifting, transferring, and repositioning, contributing to high rates of work-related musculoskeletal disorders [5, 7, 8]. These exposures represent a persistent challenge for healthcare organisations, with implications for worker health, work ability, and sustainability of the healthcare workforce [9, 10].

Various interventions targeting physical exposure during PHM have been developed to address these challenges. Studies have evaluated training programs, ergonomic equipment, and organisational strategies, examining outcomes such as musculoskeletal pain, injury incidence, sickness absence, and safety climate [6, 11]. Systematic reviews suggest that single-component interventions have limited effectiveness, whereas multifactorial approaches are more likely to influence workplace safety, but more high-quality randomised controlled studies are needed [6, 11].

In the USA, legislation governing safe PHM has several interrelated components, requiring hospitals to implement policies and programs for safe PHM [12]. According to cross-sectional findings, the legislation has led to improvements in nurses’ awareness of their hospital’s policy on safe PHM, receipt of annual training in safe PHM, and availability of lift equipment, along with reductions in the prevalence of work-related musculoskeletal symptoms [13]. However, limited use of work equipment and perceptions of persistent high injury risks indicate sub-optimal implementation of safe PHM programs [13–15]. Despite these findings, little is known how safe PHM programs are enacted in practice and the factors that influence their implementation. Further research evaluating the effect and implementation of multifactorial interventions promoting safe PHM is therefore warranted [16]. To remove barriers and support safe patient handling practices, interventions need to involve employees, enhance organisational support, foster a robust safety culture, and ensure that work equipment is available [14]. Creating a sustainable safety culture requires a systems perspective that recognises the complex interactions within healthcare organisations [17].

In line with this evidence base and research gap, a multifactorial safe PHM intervention was developed and evaluated within a cluster randomised controlled trial (RCT) in Swedish healthcare [18]. The intervention was grounded in Swedish national recommendations for safe patient handling [19], which are based on a systems perspective that recognizes how different components of the work system interact with and influence care processes, which in turn affect outcomes related to both patient safety and HCWs’ safety and health [17]. The multifactorial intervention integrated structured risk assessments, access to appropriate patient handling equipment, a participatory approach, and educational activities aimed at strengthening safety practices at the organisational level [18]. The intervention was implemented using an implementation strategy that emphasised local leadership involvement [20, 21] and facilitation [21] by internal implementation teams supported by external facilitators for integration into existing workplace practices [22]. The trial was designed as a hybrid evaluation, explicitly recognising the importance of assessing both intervention effectiveness and implementation processes [18].

While RCTs are essential for examining clinical intervention outcomes, they provide limited insight into how complex interventions are interpreted, enacted, and integrated into everyday clinical practice across diverse organisational contexts [23]. Implementation research is the scientific study of methods to promote the systematic uptake of research findings and other evidence-based practices into routine practice, and hence to improve the quality and effectiveness of health services [24]. The implementation of an evidence-based intervention is shaped by the influence of context, and understanding what happens when the intervention is integrated into clinical practice is important to better address implementation challenges in healthcare [25–27]. Implementation outcomes, defined as “the effects of deliberate and purposive actions to implement new treatments, practices, and services” [26], are therefore considered distinct from clinical and service system outcomes.

Little is known about how multifactorial PHM interventions are enacted in practice, and there is limited empirical insight into how implementation strategies lead to integration of safe PHM practices in routine care. By focusing on stakeholders directly involved in implementation following a cluster RCT, this study provides insight into the enactment of a multifactorial PHM intervention in practice and how the implementation strategy may facilitate its uptake and integration into routine care. Accordingly, this study reports a retrospective implementation evaluation conducted following a large cluster RCT [18], using individual interviews with the implementation stakeholders: healthcare managers, implementation team members, and external facilitators. The aim was to explore these stakeholders’ experiences of implementing a multifactorial intervention for safe PHM. The research questions were:

What determinants influenced the implementation and sustained use of a multifactorial intervention for safe PHM in clinical practice?

How are the actual implementation outcomes perceived and described by the implementation stakeholders?

## Methods

### Study design and setting

This study employed a qualitative explorative and descriptive research design using semi-structured interviews [28]. The study is part of a research project with a prospective cluster RCT evaluating the implementation and effectiveness of interventions for safe PHM. The RCT is based on a hybrid 2 trial design, where clinical effectiveness and implementation outcomes are given equal priority [18]. The primary outcome of the trial is occupational safety climate measured with questionnaires. In this study, we interviewed stakeholders about their experiences of the implementation to explore the process evaluation components implementation, context, mechanisms of impact and outcomes [23]. The clinical effectiveness, implementation outcomes and what is delivered, such as fidelity, will be described elsewhere. The RCT was conducted in 14 Swedish regions, including a total of 46 healthcare units from hospitals and homecare settings. These units were stratified and randomised to a multifactorial (*n* = 23) or single intervention strategy (*n* = 23). Units in the multifactorial intervention fulfilling the 12-month follow-up were eligible to participate in this qualitative study (*n* = 14).

### Clinical intervention

The multifactorial intervention is described in detail elsewhere [18]. Briefly, it included risk assessments, access to equipment, theoretical and practical education, and organisational strategies (e.g. leadership engagement and work environment mapping) to support safe PHM practices [29] (Table 1 ). It was based on the Swedish research-based guide for safe PHM practices [19], integrated structured risk assessments with the TilThermometer to assess HCWs’ physical workload [30–32], and the Downton Fall Risk Index to identify patients’ fall risk [33]. In the TilThermometer, the HCWs’ risk for physical exposure is assessed in five different types of PHM situations in relation to the care recipients’ function and level of activity [30]. The Downton Fall Risk Index is a screening tool used to identify individuals at increased risk of falls. The instrument assesses previous falls, medication use, sensory impairments, cognitive status, and gait ability, yielding a total score between 0 and 11, where scores of 3 or higher indicate elevated fall risk [33]. A digital introduction to the research-based guide was included along with digital modules providing theoretical education on occupational safety and health, risk assessments, and interventions promoting safe PHM. Hands-on practical education in safe PHM was strongly advised, but not provided by the intervention itself. Instead, the intervention included recommendations regarding content and minimum scope, and the individual units were left to organise and deliver the practical education based on their local conditions.

Two participatory workshops were held with the implementation team in each unit, the external facilitator, and one of the researchers. These workshops focused on identifying and analysing risks in relation to safe PHM, and jointly developing a unit-specific action plan containing measures to address the risks and improve the working environment, ensuring that the interventions were tailored to the unit’s context.

### Implementation strategy

The implementation strategy (Table 1; see also [18]) was based on facilitation [21] by using internal implementation teams supported by external facilitators [22]. Mid-level leaders were expected to indirectly lead their unit’s implementation of the safe PHM practice via implementation teams comprising approximately 4–8 unit personnel. These teams were responsible for implementing and tailoring the safe PHM practice to their unit’s specific context, supported by the leader and an external facilitator. The external facilitator guided and supported the team, addressed context-specific challenges, and promoted adherence to the intervention and study procedures. The research team provided support throughout the study period.

**Table 1:** Components of the multifactorial safe patient handling and movement (PHM) intervention and the implementation strategy

Multifactorial safe PHM intervention	Implementation strategy
Research-based guide (80 pages)	Internal facilitation: implementation teams, meetings every second week, work according to steps in the guide
Introduction (23 min)	External facilitators: supporting managers and teams
Digital theoretical education (2 × 35 min)	Information, ongoing support and reinforcement strategies
Practical hands-on training (1 day^*^)	Audit and feedback (continuing)
Assessment of working environment and feedback (questionnaire)	
Participatory workshops^#^ (2 × 2 h)	
Risk assessment:	
• HCWs’ physical workload (4 times^*^)	
• Patients’ fall risk (4 times^*^)	

^*^ Minimum recommendations; ^#^ Manager, implementation team members, and external facilitator participating, led by external facilitator or one of the researchers; HCW, healthcare worker

### Participants

A purposive sampling strategy was conducted to ensure a variety of implementation stakeholders from both hospitals and nursing home units and from different regions. Participants represented a range of roles and settings, enabling exploration of diverse implementation experiences. We aimed to interview the manager and at least one implementation team member from each unit. At the beginning of 2024, the research team approached eligible participants (14 managers, 14 implementation teams, and seven external facilitators) by e-mail, including information about the study and conditions for participation. Nine managers, ten implementation team members, and six external facilitators agreed to participate and provided their signed written informed consent. Participant characteristics are presented in Table 2.

**Table 2:** Characteristics of participants in individual interviews (*n* = 25)

	Managers (*n* = 9)	External facilitators (*n* = 6)	Implementation team members (*n* = 10)
**Length of interview**, minutes, mean (range)	43 (27–67)	50 (43–60)	36 (17–54)
**Age**, mean (range)	48 (30–63)	48 (36–63)	45 (28–66)
**Sex**, n, women/men	9/0	5/1	8/2
**Profession**,** n**			
Licensed practice nurse	5	1	-
Assistant nurse	2	1	8
Physiotherapist	-	2	1
Occupational therapist	-	2	1
Social worker	2	-	-
**Working experience**, years, mean (range)	23 (9–34)	18 (9–28) *	23 (2–48)
**Hospital/community unit**, n	6/3	3/3	6/4

*Information on years of experience was missing for one participant

### Data collection

A semi-structured interview guide (Additional file 1) was developed iteratively by the authors (LW, ENS) based on the study aim, previous literature, and prior experience from the trial. The main interview questions concerned perceptions of the content and practical gain of the multifaceted intervention for safe PHM, and experiences of the implementation strategy with a focus on the stakeholder role and determinants for the implementation. Probes were used to deepen the answers. Individual interviews were conducted during February–April 2024, either digitally via the Visiba Care platform (*n* = 9) or face to face (*n* = 16). The interviewers (CW and ENS) were involved in the main research project but had no previous relationship with any of the informants. The interviews lasted 17–67 min (mean 41 min), were digitally recorded and transcribed verbatim by a professional transcriber. Transcripts were checked for consistency and pseudonymised before being imported into NVivo (Lumivero, version 14) for management and coding of the data.

### Data analysis

Data were analysed using qualitative content analysis with a deductive approach [34], guided by the Consolidated Framework for Implementation Research (CFIR) [27, 35, 36]. This is a determinant framework including constructs from several implementation theories, models, and frameworks, used to predict or explain barriers and facilitators to implementation success. The updated CFIR contains 48 constructs and 19 subconstructs across 5 domains [35], and an outcomes addendum has recently been proposed [37]. The data were first inductively coded to capture the participants’ experiences, followed by deductive mapping of codes [34] to the CFIR [27, 35–37]. ENS and CW read the transcripts several times to become immersed in the data and obtain a sense of the whole. The inductive coding was conducted by ENS, based on the content and directed by the study aim and research questions; this was followed by continuous discussions with CW, who also critically read the codes. Next, the analysis was conducted using a deductive approach, with ENS being mainly responsible. An unconstrained categorisation matrix (Additional file 2) was developed on the basis of the CFIR [27, 35–37]. The matrix’s first three columns are based on the CFIR coding guidelines [36], whereas the final column contains study-specific coding guidance developed to further enhance the dependability of the analysis (Additional file 2). The codes were reviewed for content and mapped into the domains and constructs of the CFIR. Categories within the bounds of CFIR constructs were developed inductively. The analytical process and tentative categories were repeatedly discussed among all the authors until consensus was reached on the CFIR constructs, inductive categories, labels, and illustrative quotations [38].

The research team comprised healthcare professionals with experience of clinical work in Swedish hospitals and elderly care, and hence an insider perspective. However, one researcher was an outsider to the Swedish healthcare context. All authors were part of the main research project, and had varied empirical and scientific knowledge of safe PHM and implementation science. All authors were experienced qualitative researchers within healthcare research, but had diverse backgrounds regarding profession (physiotherapist/occupational therapist), age, gender, country, and academic experience. These different perspectives helped to raise reflexive questions on relationships, interactions, power dynamics, and assumptions during the research process. To enhance credibility, the categorisation, labelling of categories, and selection of quotations were discussed among the authors. Reflexive discussions were held throughout the analysis process to challenge interpretations and assumptions. A reflexive statement is provided in Additional file 3.

### Ethical approval

Permission to conduct this study was granted by the Swedish Ethical Review Authority (ref: 2021–00578). Written informed consent was collected from all participants. The study followed the ethical principles for medical research stated in the Declaration of Helsinki [39] and was reported in line with the criteria for reporting qualitative research [40] and the StaRI checklist [41].

## Results

The analysis of the interviews resulted in deductive categories based on the CFIR (five domains and nine constructs) and 13 inductively-developed categories describing determinants influencing the implementation of a safe PHM practice (Table 3). In addition, the participants described implementation outcomes reflecting the implementation, enactment of the intervention in practice, and conditions for sustained use. These outcomes were closely linked to changes in safety culture, work routines, and use of patient handling equipment.

**Table 3:** Consolidated Framework for Implementation Research (CFIR) domains and constructs, and inductively generated categories

Inductive category	Subcategory (CFIR construct)	Main category (CFIR domain)
Intervention welcomed but its complexity required time for embedding into practice	Innovation design	Innovation
Benefits of structured risk assessments incorporated in daily practice	Innovation relative advantage	
Enacting the safe PHM practice by tailoring theory and practice to context and needs	Access to knowledge and information	Inner setting
Influence of unit conditions on practical education and work equipment	Available resources	
Leadership promoting change in PHM practice	Implementation leads	Individuals
Facilitators’ personal capabilities and competencies enabling implementation support	Implementation facilitators	
Facilitators’ supporting and driving role		
Barriers and opportunities to enact facilitation		
Driving and engaging capabilities of interprofessional teams	Implementation team members	
Opportunities for authority and team consistency for implementation teams		
Facilitators’ support and implementation teams’ guarding of safe PHM practice	Doing	Implementation process
Change in PHM safety culture and practice	Implementation sustainment	Actual implementation outcome
Sustainment of safe PHM practices		

### Innovation

#### Innovation design

The informants *welcomed the intervention*,* but its complexity required time for embedding into practice*. They appreciated its structured design and the inclusion of multiple components. However, it was initially perceived as complex and challenging to integrate into existing workflows. Extending the implementation period from 4 to 12 months facilitated the embedding of safe PHM into routine practice. The research-based guide was valued for its practical content and structured checklists, which provided hands-on guidance. However, several participants found it difficult to navigate, and suggested a shorter, more accessible version of the guide to support wider use among HCWs.

*I think it’s been really good. We don’t have experience with other interventions*,* but we’ve talked about it a bit and feel that this one worked well for us. It also feels like we’ve been able to work with it in a good way*. (Team member, 5)

#### Innovation relative advantage

*Structured risk assessments were beneficial*, and perceived as a key improvement over previous practice. The TilThermometer provided a clearer and more visual understanding of physical workload, supporting team-based discussions and more informed decisions regarding equipment use. While some challenges were noted in dynamic patient contexts, the tool increased awareness of workload and facilitated shared decision-making across professions.

*And it’s not just the actual part of putting on the compression stocking. It’s everything around it*,* it’s hard — you have to bend down*,* get on your knees. And then I ask*,* why do you have to do that? And they say*,* “Well*,* they can’t get it onto their foot.” And you’re like*,* “But they could be lying in bed?” So it becomes more of a whole discussion around it — “Oh*,* right*,*” you start thinking*,* and so on. And these were the kinds of things we talked about at our workplace meetings; it made everything more concrete*,* more real*,* more about finding solutions*. (Leader, 2)

### Inner setting

#### Access to knowledge and information

The safe PHM practice was described as being *enacted by tailoring theory and practice to context and needs.* Theoretical knowledge on safe PHM practices was accessible via videos that were available during the entire study period. These videos were generally described as informative, but not all of them were relevant to all units, as transfer situations and patient mobility levels differed between units. In some of these cases, the manager selected videos that were relevant to the unit. Some HCWs watched and discussed the content of the videos in groups over a couple of weeks, for instance during workplace meetings or educational days. They thought that this increased their knowledge to a greater extent than if they had watched the videos individually. Discussions on the applicability within the unit were highly appreciated and described as enabling learning, particularly when HCWs had language difficulties. In one unit, the videos were presented as a shared activity to motivate engagement.

Practical education in safe PHM was considered crucial and highly valued. Although not directly delivered by the intervention, it was organised locally and therefore varied in content and duration. The HCWs described how practical education supported learning for both new and experienced staff. Organisational learning was particularly enabled when education was conducted collaboratively, for example during workplace meetings or when the staff participated together as a team.

*I really think it was very helpful. This is what we mean when we talk about competence — we actually strengthened their competence. And before*,* in this municipality*,* they’d send three or four people for training at a time*,* and then another three later on. But if you want to build some kind of group*,* a group culture around safe transfers — good transfers for both the users and the staff — then the whole group needs to be involved. And that was the opportunity we had*,* and I know the staff really appreciated it.* (Team member, 6)

#### Available resources

*Conditions within each unit influenced both the practical education and the work equipment.* The setting and procedures of the practical education therefore differed between the participating units, which affected their possibility to educate their HCWs. A few units had “in-house” practical-education programs for safe PHM, which made it easier for the HCWs to attend the education, while others arranged the education locally using physiotherapists, occupational therapists, or the occupational health service. Others had to send their employees to external educators, which had financial consequences for the unit. The managers decided whether only new HCWs, a few HCWs, or all HCWs should participate in the practical education, resulting in variation in the reach of education between the participating units. A collective understanding was that all HCWs should participate in order to share the same knowledge base and learn from each other, which would facilitate safe PHM practices.

Conditions within the inner setting could also hamper the use of work equipment, such as when the physical working environment could not be changed, or when financial constraints hindered purchasing new equipment. Procurement of work equipment was described as challenging; for example, in many units, ceiling lifts were considered too expensive to order. This was expressed as a struggle, and the participants highlighted the need for clearer responsibility and mandate for procurement of work equipment. Ongoing organisational constraints, including staffing limitations and staff turnover, made it challenging for managers to consistently prioritise safe PHM practice. Scheduling issues and weak collaboration between nursing personnel and allied HCWs were also barriers.

*Well*,* I mean*,* generally I think it’s like this: it’s not just about having a guideline that says you should get training. It’s also about asking*,* “What effect does this actually have on patient safety? What effect does it have on the work environment?” It’s as if the organisation doesn’t really understand how important this is. So I think a guideline*,* or a routine*,* or an action plan — especially at the regional level — really needs to exist and be anchored properly so it actually gets prioritised.* (Facilitator, 1)

### Individuals

#### Implementation leads

The informants described how *leadership could promote change in PHM practice.* Those of them who were leaders themselves emphasised the importance of implementing safe PHM practices for patient and employee health and safety, and for highlighting risks in female-dominated working environments. Having a full year dedicated to the implementation enabled them to prioritise safe PHM and support meaningful changes in practice. The collaborative structure, including the implementation team and external facilitator, helped maintain momentum despite organisational constraints.

Leaders were described as central to the implementation, providing structure, motivation, and direction. By offering support and opportunities, they enabled implementation team members to fulfil their roles. Team members described feeling trust from their managers and being given the mandate to make changes. Crucial aspects included engagement among the leadership, involving staff, and demonstrating that the work was important. Leaders also took responsibility for strategic communication within and outside the unit to reinforce motivation and sustain the work.

*What the managers shared was that they genuinely prioritised this and made it very clear: “This matters. You matter. Your work environment is really important*,* and that’s why we’re doing this for you.” And I think that’s really*,* really important.* (Facilitator,1)

#### Implementation facilitators

Several of the *facilitators’ personal capabilities and competencies* were considered important for the role of implementation facilitator. Being interested in quality improvement work and safe PHM practices was a valuable characteristic. Experiences of previous quality improvement work or project leading were highlighted, as was local knowledge about the unit, its patients, and its working environment that could help the facilitator to understand the unit’s needs and tailor the support accordingly. Competence in listening, coaching, and guiding was also valued.

*I’ve tried to read up on the method itself*,* and also*,* based on what the clinic or the unit wanted*,* to figure out how much I should be involved and what I could contribute.* (Facilitator, 3)

The facilitators described their role as *coordinating*,* supporting*,* and driving* the process forward. Depending on the unit’s need and the engagement of the implementation team and leader, the support varied from practical, hands‑on assistance to being a sounding board.

*I guess you could say I’ve been the project leader for this unit — inviting people to meetings*,* trying to run the meetings*,* taking notes*,* keeping all the information together*,* and suggesting what we should do at each meeting.* (Facilitator, 6)

The facilitators described *barriers and opportunities for enacting facilitation*, such as the challenge of fulfilling the mission within their ordinary working tasks, especially when the unit was not located nearby. They found it relatively easy to contact the leaders, but not always so easy to contact the implementation team members. The facilitators said it was easier to support the units that had well-functioning implementation teams with an appointed leader, while it could be difficult to even reach other implementation teams.

*I’ve done all of this alongside my regular duties*,* so I’ve been doing it in time that I didn’t really have. And I haven’t been out at the care homes as much as may have been originally intended for a facilitator.* (Facilitator, 4)

#### Implementation team members

Members of the implementation teams had a variety of professions; they mainly consisted of nursing personnel, but sometimes allied professionals were also involved, which was considered preferable. These *interprofessional implementation teams were described as having driving and engaging capabilities.* The participants highlighted the importance of the team members being interested in safe PHM issues, prioritising the work, and having competence to collaborate with each other to drive the work forward and engage all staff at the unit.

*And it’s also been the case that those of us who remained have been the driving force. If we hadn’t taken the lead*,* things wouldn’t have changed much. And we’ve brought it up with the whole larger team*,* not just kept it within the implementation group.* (Team member,1)

The implementation teams also required *opportunities for authority and team consistency.* One leader stated that the role of the implementation team facilitated unit-specific ownership of the implementation work, and having a consistent implementation team was expressed as a “winning formula”. However, staff turnover in the implementation teams and among allied professionals was a challenge, making it difficult to sustain the work and engage the staff. Overall, leaders were described as having provided support in terms of permission to set aside time for the work, change schedule, and provide trust to the team. This was considered imperative to succeed with the implementation.

*I mean*,* we were given time set aside for this*,* and that’s really been the key*,* I think. In a small and unstable organisation*,* that’s probably been the biggest factor.* (Leader, 7)

### Implementation process

#### Doing

*Support from a facilitator was important for the implementation teams*, but the need varied depending on the capabilities of the implementation team members. Some teams worked independently according to the steps in the research-based guide, but most of the teams required practical support from the facilitators in how to use the guide and follow the steps. Regularly scheduled meetings with the implementation teams and leaders, which included giving the teams missions and setting goals, carried the process forward. The facilitators introduced the teams to risk assessments with the TilThermometer; their participation varied from ensuring that the risk assessments were performed to participating in all risk assessments and summarising the results. The development of an action plan was especially important and helpful to follow.

Facilitators maintained ongoing dialogue with leaders in order to align the work with unit needs and priorities. Implementation teams described taking responsibility for maintaining a focus on safe PHM at their units, using workplace meetings and other strategies to engage staff.

*Yeah*,* I think each of us four who’ve been involved has really taken responsibility. We’ve tried to get everyone on board and bring it up at our staff meetings — how important it is and how we should think about it. (…) and really tried to say*,* “Let’s do it this way*,* and remember to think about your body.”* (Team member, 10)

### Actual implementation outcomes

#### Implementation

Participating in the intervention meant that the focus was on safe PHM practices and the HCWs perceived that a *change in PHM safety culture and practice* was enabled. The HCWs started to talk about PHM both between themselves and with allied professionals. They discussed patient handling situations with a new awareness, and paid attention to risks. The managers noted that the HCWs were now working more systematically with PHM issues, including when introducing new staff. It had become easier to remind each other how to work, and to explain the procedures to patients and their relatives.

*I think this has really brought attention to the issue in a completely different way. People now understand that patient transfers are a clear part of care work. Before*,* it was maybe just something you did*,* but now you realise it’s something that requires attention and a systematic way of working. So that’s the benefit it’s brought.* (Facilitator, 3)

Participating in the intervention also influenced HCWs’ perceptions of work equipment. Risk assessments were described as “eye-opening” for identifying equipment needs. Routines were established for work equipment, checklists were provided and followed, and HCWs responsible for work equipment were selected. The HCWs talked more about work equipment after the implementation, and dialogues were held within the team and with allied HCWs, covering what to use and when.

*What really impresses me is that now*,* when I walk down the corridor*,* I can see that people don’t just go in and do a transfer anymore. They actually stop and talk first: “What equipment do we have in the room? What do we need? Who needs to be involved?” And I also heard something that really warmed my heart — someone said*,* “Which one of us gets the patient involved?” Because it’s no good if everyone talks at once. So they decided beforehand: “Can you go in and talk to the patient about the transfer? And then we’ll come in*,*” because there needed to be several people involved. And that made me realise how much things have changed*,* because that’s not how we used to work — we just went straight in.* (Leader, 6)

#### Sustainment

The participants expressed a clear ambition *to sustain the safe PHM practice that had been implemented*. Implementation team members and managers said that they planned to continue using the TilThermometer and the Downton Fall Risk Index, to establish routines, and to spread the practice to other units. Preconditions for sustaining the safe PHM practice included a strong work infrastructure with regular education, structures for introducing and educating new employees, and networks of peer coaches to provide training in work equipment and safe PHM. Leadership support at different manager levels was described as crucial for sustainment and upscaling, alongside HCWs’ engagement and collaboration with allied professionals. Maintaining competencies in the implementation teams was highlighted, as there was a need for someone to lead the work. Several informants emphasised the need to incorporate safe PHM issues such as education within annual health and safety work cycle, and to develop practical guidelines pointing out directions for organisations.

*I think that’s really important — that leadership treats it as a key issue. And I know [name] is working on a strategy now*,* which is hugely important*,* having something overarching. And I know things are moving forward.* (Facilitator, 6)

## Discussion

This study identifies key determinants and perceived outcomes influencing the implementation of a multifactorial intervention for safe PHM practices. Interdependence between leadership, facilitation, and stakeholders’ active engagement with knowledge about safe PHM shaped the implementation process, enabling translation into practice. Implementation outcomes were reflected in the perceived integration of safe PHM practices into clinical routines and changes in safety culture. These findings also highlight the importance of organisational and collaborative processes in supporting the sustained use of new practices. From a systems perspective, the findings illustrate how different components of the work system (e.g., person, tasks, and tools) influenced PHM practices (care processes) and, in turn, affected outcomes related to both patient safety and HCWs’ safety and health. The proposed relationships between determinants, strategies, intervention, mechanisms of impact and outcomes are shown in an implementation research logic model [23, 42] presented in additional file 4.

Leadership was a central driver of the implementation process. Leaders were not only supportive, but actively created the conditions necessary for implementation, including prioritisation of safe PHM, allocation of sufficient time, and provision of structure. A dedicated implementation period enabled leaders to focus on the work and support meaningful changes in practice, while the collaborative structure involving implementation teams and external facilitators helped sustain momentum despite organisational constraints. The importance of leadership in facilitating implementation of evidence-based practices is well established [21, 27, 43–46]. Leadership engagement is also emphasised in participatory ergonomic interventions to allow for a positive implementation climate, organisational changes and resource allocation [47]. Our findings mirror and extend this understanding by illustrating how leadership operates as a key mechanism enabling implementation teams to act by providing trust and opportunities to make changes, supporting staff engagement and collective responsibility for the work. These findings align with implementation research emphasising leadership as critical for embedding evidence-based practices through shaping organisational priorities and reinforcing the importance of the intervention [45, 48].

Recent evidence suggests that the influence of leadership lies in how leaders create implementation conditions through commitment, supportive relationships, and building learning capacity [48–50]. Our findings extend this by showing how leaders provided mandate, time, structure, and trust within safe PHM implementation. However, leadership alone was not sufficient, as its influence depended on the outer context, such as higher-level managers and broader organisational conditions, as well as on the presence of functioning teams and facilitation. Several of the identified determinants (i.e., leadership, team, setting) may reflect how the different components in a work system interact, and thereby help to clarify the mechanisms through which the reported changes in safety culture may have emerged [17]. Together, these findings indicate that leadership drives implementation by creating the conditions for action, rather than acting solely as a source of support. Further research is warranted on higher-manager levels, as these shape the conditions for units to purchase equipment, fund staff education, and combine occupational safety and health with patient safety.

Internal facilitators working in teams are key to knowledge implementation, but role clarification, support, preparation, and contextual understanding are important [51]. Our findings support recent work suggesting that facilitation is an active, relational, and context-sensitive process. As depicted in the i-PARIHS framework, successful implementation results from the facilitation of an innovation with the intended recipients in their contextual setting [21]. The external facilitators supported coordination and problem-solving, but their influence depended on engaged implementation teams, leadership, and local organisational conditions. In units with stable teams and engaged leaders, facilitation catalysed progress, whereas organisational constraints limited its influence. Similarly, Connolly et al. highlighted the importance of considering the role of environmental factors during facilitation and how the processes and interactions between external and internal facilitators occurred more smoothly with cohesive and well-established teams [52]. These findings suggest that facilitation is not a standalone driver of implementation, but a context-dependent process that supports implementation when combined with leadership and functioning teams.

The clinical intervention for safe PHM included the use of videos and practical training to increase knowledge. Education on safe PHM appeared to function as a starting point for collective learning, demonstrating that knowledge only becomes effective when it is actively taken up, adapted, and owned by participants within their local context. This is consistent with previous research showing the role of collective workplace learning for shared understanding and supporting the translation of knowledge into practice; local adaptations may reflect participants’ ownership of both content and process [53]. For example, Eriksen et al. evaluated an educational intervention to implement video consultations in two Danish hospitals. Formal education alone was insufficient, but informal workplace learning, local adjustment, and support in practice improved HCWs’ skills [54].

Our study also found that collective discussions appeared to support shared understanding and ownership of safe PHM practices and an awareness that safe PHM practices also include the safety of HCWs. An interesting finding was the role of the TilThermometer, which appeared to function as more than a risk assessment tool. Rather than solely identifying patient handling risks, it served as a practical and active mechanism for implementation by making the physical workload visible, creating a shared language among staff, and facilitating discussions about patient handling decisions and equipment use. The tool thus supported collective reflection and contributed to translating knowledge of safe PHM into everyday practice. This finding suggests that assessment tools may influence practice not only through their technical function but also through their capacity to support communication, shared understanding, and team-based decision-making. Successful implementation depended not only on access to knowledge, but on the way in which knowledge and tools were collectively used, adapted, and owned in practice. These findings may resonate with the theory of communities of practice, which describes how groups of people who share a common concern or a passion for a topic deepen their knowledge and expertise through ongoing interaction. Further research is needed to assess whether applying this theory can improve clinical outcomes and facilitate the sharing of vital information across an increasingly complex healthcare system [55],

Another contribution of this study is the identification of early signs of sustainment following implementation. The participants described changes in safety culture, routines, sustained use of risk assessments, and patient handling equipment, indicating that safe PHM practices were integrated into everyday work and new norms had been internalised. This integration into practice appeared to be supported by leadership engagement, ongoing training structures, and collective responsibility within teams. Our findings align with sustainability literature emphasising organisational infrastructure, fit with local context, and ongoing resources as key conditions for maintaining interventions [56, 57]. This also highlights the importance of considering sustainment as an integral part of implementation, and the need for system-level conditions to support lasting change.

Regarding clinical implications, the present findings suggest that organisations seeking to improve patient handling safety should focus on a collaborative approach, creating the conditions for collective learning and routine integration. Standardised tools for risk assessments provide structure and go beyond risk identification towards a common understanding for workplace safety. Longitudinal studies are needed to explore how leadership support, implementation team continuity, and facilitation influence the sustainability of safe PHM practices beyond the trial period.

### Methodological considerations

The strengths of this study include the purposeful sampling strategy, the adequate sample size, and the variation of participant characteristics. Data were collected and analysed by experienced qualitative researchers. Continuing discussions among the authors were held for triangulation, enhancing credibility. Dependability was improved by using the CFIR as a theoretical framework for the structured deductive analysis, in line with previous work [36]. Culture is a construct defined as the shared values, beliefs, and norms across the domain Inner setting [35], however, in this study we analysed and interpreted the descriptions of changes in safety culture as actual implementation outcomes [37]. The relations between implementation determinants, interventions, mechanisms and outcomes are outlined in a logic model (Additional file 4). The interviews were conducted within three months after ending the RCT, which reduced the risk of recall bias.

This study also has limitations. Participants were recruited from an RCT, and only participants from units that completed the 12-month follow-up were eligible for this qualitative study, potentially favouring more engaged units or units with more successful implementation. Hence, these participants may have been more interested or motivated in changing to a safe PHM practice than others, hampering transferability. However, both positive and negative experiences were described in the interviews. No member check was performed, and transcripts were not returned to the participants; doing this could have strengthened the analytical process. To avoid the risk of favourable interpretations due to our preconceptions and roles, emphasis was put on the participants’ descriptions, focusing on the manifest content; moreover, we continued to discuss our preconceptions throughout the research process. The findings from this qualitative study need to be complemented with the outcomes from the RCT to gain a better understanding of what may contribute to a safer PHM practice.

## Conclusions

This study explored stakeholders’ experiences of implementing a multifactorial intervention for safe PHM, identifying key determinants and perceived outcomes influencing its integration and sustained use in clinical practice. The interdependence between leadership, facilitation, and participants’ active engagement with and ownership of knowledge were central determinants shaping the implementation process for workplace learning. These factors may have contributed to the translation of knowledge into practice, supporting changes in routines and safety culture. Implementation outcomes were reflected in the perceived integration of practices into everyday work, indicating early sustainment. Overall, the findings indicate that implementation success may depend on organisational and collaborative processes that support the sustained use and embedding of new practices.

## Supplementary Information

Below is the link to the electronic supplementary material.

Supplementary Material 1

Supplementary Material 2

Supplementary Material 3

Supplementary Material 4

## Data Availability

The data that support the findings of this study are not openly available due to reasons of sensitivity and are available from the corresponding author upon reasonable request.
