# Supplementary material for: Stakeholders’ experiences of the implementation of a multifactorial intervention for safe patient handling and movement: a qualitative explorative and descriptive study
Source: BMC Health Serv Res. 2026 Nov 6;26:1545. doi: 10.1186/s12913-026-16068-8 (PMC13741535; doi:10.1186/s12913-026-16068-8)
Supplement: Supplementary file 1 [file 12913_2026_16068_MOESM1_ESM.docx]

**Interview guide**

1. Introduction question: You work as… Please briefly describe your work role and tasks.

2. How do you perceive the content of the multifaceted intervention for safe patient handling and movement (PHM)?

- The guide, risk assessments, action plan, education (theoretical and practical training)

3. What improvements, if any, has the implementation of the intervention brought for your care unit?

4. What do you think of the strategy for implementation of this intervention for safe PHM?

- What was good? What could have been better?

5. You had a special mission within this implementation strategy. Could you describe your role and tasks?

- What has enabled the work? What barriers have you encountered? Which strategies have you/the team used? Was anything particularly applicable/useful?

6. What do you think about the unit’s continued work with safe PHM in the future?

- What is needed for you to continue the work?

7. Ending question: Is there anything else you would like to talk about that we haven’t discussed?
