# Supplementary material for: Stakeholders’ experiences of the implementation of a multifactorial intervention for safe patient handling and movement: a qualitative explorative and descriptive study
Source: BMC Health Serv Res. 2026 Nov 6;26:1545. doi: 10.1186/s12913-026-16068-8 (PMC13741535; doi:10.1186/s12913-026-16068-8)
Supplement: Supplementary file 2 [file 12913_2026_16068_MOESM2_ESM.docx]

# CFIR code book, based on CFIR coding guideline by Reardon et al. 2025 References

# Readon, C.M., Damschroder, L.J., Ashcraft, L.E. et al. The Consolidated Framework for Implementation Research (CFIR) User Guide: a five-step guide for conducting implementation research using the framework. Implementation Sci 20, 39 (2025). <https://doi.org/10.1186/s13012-025-01450-7>

## Innovation Domain Coding

| **I. INNOVATION DOMAIN** | ***Innovation:*** The “thing” being implemented [2], e.g., a new clinical treatment, educational program, or city service.  ***Project Innovation:*** [Document the innovation being implemented, e.g., innovation type, innovation core vs. adaptable components, using a published reporting guideline [3], [4], [5], [6]. Distinguish the innovation (the “thing” that continues when implementation is complete) [2], [7] from the implementation process and strategies used to implement the innovation [8], [9] (activities that end after implementation is complete) [10].]  ***Note:*** *Many constructs in the Innovation Domain may be more relevant pre-adoption of the innovation (when key decision-makers are deciding whether to put the innovation in place or not)* [11] *versus implementation or sustainment of an innovation.* | | ***Study specific*** |
| --- | --- | --- | --- |
| **CFIR Construct Name** | **Construct Definition** *The degree to which:* | **Coding Guidelines**  These constructs are specific to the innovation itself, capturing characteristics of the innovation regardless of context (where it is implemented) or implementation strategy (how it is implemented). As a result, teams must define the Innovation to code accurately.  When coding these constructs post-implementation, be careful to ensure the statement reflects an implementation determinant, not an implementation outcome. See coding guidelines for Innovation Evidence-Base as an example that shows this important distinction.  Add constructs to capture additional Innovation characteristics not included in CFIR. | The intervention safe patient handling and movement (PHM), its components and characteristics |
| A. Innovation Source | The group that developed and/or visibly sponsored use of the innovation is reputable, credible, and/or trustable. | Include statements about:   - The type of innovation source, e.g., external sources, including academic, governmental, or commercial entities; internal sources, including individuals or groups in the Inner Setting; and/or external/internal sources, where the innovation was co-developed and/or co-sponsored. - Characteristics of the innovation source, including but not limited to whether they are reputable, credible, and/or trustable.   Exclude statements about:   - Implementation support and/or facilitation that may be provided by the Innovation Source, and instead code Individuals Domain: Implementation Facilitators or another appropriate Role. - The innovation’s evidence-base, and instead code Innovation Domain: Innovation Evidence-Base. | Statements about the innovation source |
| B. Innovation Evidence-Base | The innovation has robust evidence supporting its effectiveness. | Include statements about:   - The presence (or absence) of preexisting evidence demonstrating that the innovation will or will not be effective. - Different types and sources of evidence, e.g., published literature, anecdotal stories from colleagues, information from a competitor, previous experiences with recipients or from a pilot, community lived experience, or indigenous ways of knowing, as well as a need for additional or different kinds of evidence.   Exclude statements about:   - Actual innovation outcomes (post-implementation) in the Inner Setting e.g., in a retrospective evaluation, a statement such as “The innovation was effective for our patients,” and instead code Innovation Outcomes: Recipient Impact [11]. - Sharing evidence to engage individuals in implementation and/or delivery of the innovation, and instead code Implementation Process Domain: Engaging. | Statements about the evidence demonstrating that safe PHM is effective |
| C. Innovation Relative Advantage | The innovation is better than other available innovations or current practice. | Include statements about:   - Various types of advantages/disadvantages, e.g., at the system-level (e.g., financially (dis)advantageous to the Inner Setting) or individual-level (e.g., clinically (dis)advantageous for deliverers/recipients).   - ***Note:*** *Relative Advantage is often associated with other constructs. For example, if an innovation is worse than current practice because it does not fit with existing workflows, code both Innovation Domain: Innovation Relative Advantage and Inner Setting Domain: Compatibility; if an innovation is better than current practice because it meets recipient needs, code both Innovation Domain: Innovation Relative Advantage and Individuals Domain: Innovation Recipients * Need.*   Exclude statements about:   - The extent to which the innovation is needed, and instead code Inner Setting Domain: Tension for Change. | Statements about advantages/disadvantages (better or worse than current practice, fit with workflows) |
| D. Innovation Adaptability | The innovation can be modified, tailored, or refined to fit local context or needs. | ***Note:*** *This construct captures the inherent adaptability of the innovation,* ***not*** *the need to adapt nor the process of adapting the innovation.*  Include statements about:   - The (in)ability to adapt the innovation, due to features of the innovation itself, e.g., a rigid protocol or lack of “permission” to change components.   Exclude statements about:   - The process of adapting the innovation or types of adaptations that will be/were made, and instead code Implementation Process Domain: Adapting. - The innovation needing/not needing to be adapted, and instead code the relevant CFIR construct, e.g., if an innovation needs/needed to be adapted because it did not fit with existing workflows, code Inner Setting Domain: Compatibility. | (in) ability to adapt safe PHM due to features in itself |
| E. Innovation Trialability | The innovation can be tested or piloted on a small scale and undone. | ***Note:*** *This construct captures the inherent trialability and/or reversibility of the innovation,* ***not*** *the need to trial nor the process of trialing the innovation.*  Include statements about:   - The (in)ability to trial the innovation, due to features of the innovation itself, e.g., a very complex, high-cost innovation may be more difficult to trial (less reversable) than a very simple, low-cost innovation.   Exclude statements about:   - The process of trialing the innovation, and instead code Implementation Process Domain: Doing. - Use of results from pilots, and instead code Innovation Domain: Innovation Evidence-Base. | Statements on the (in)ability to trial the safe PHM intervention due to features in itself |
| F. Innovation Complexity | The innovation is complicated, which may be reflected by its scope and/or the nature and number of connections and steps. | ***Note:*** *This construct captures the complexity of the innovation,* ***not*** *the complexity of implementation of the innovation.*  Include statements about:   - The complexity or simplicity of the innovation based on its scope and/or the nature and number of connections and steps, e.g., a year-long multidisciplinary program with a lot of moving parts may be considered a complex innovation whereas exchanging one type of medication for another may be considered a simple innovation.   Exclude statements about:   - The complexity of implementation, and instead code the relevant CFIR construct, e.g., if implementation is complex due to lacking sufficient funding, it would be coded to Inner Setting Domain: Available Resource: Funding.   - ***Note:*** *If the statement is about the complexity or difficulty of implementation influencing the likelihood of implementation success, code Anticipated Implementation Outcomes: Implementability* [11]*.* | Statements on the complexity or simplicity of the safe PHM intervention (such as reading the guideline) |
| G. Innovation Design | The innovation is well designed and packaged, including how it is assembled, bundled, and presented. | Include statements about:   - Quality of the design and packaging of the innovation, e.g., flow, format, and organization of innovation components, as well as associated physical, electronic, or online materials. - The presence or absence as well as types of materials bundled (or not bundled) with the innovation including:   - Branding/marketing materials, e.g., posters.   - Recipient materials, e.g., worksheets, user interfaces.   - Deliverer materials, e.g., pocket cards.   - Implementation materials, e.g., an implementation toolkit. ***Note:*** *Implementation materials should only be coded to this construct if they are considered part of the innovation vs. implementation strategy.* Implementation materials may include information regarding:     - Costs, resources, competencies, and training specifications.     - Evidence supporting the effectiveness of the innovation.     - Recommended strategies to implement the innovation and engage deliverers and recipients.     - Instructions for deliverers, e.g., information is provided to help apply to individual recipients.     - Measures to evaluate the innovation.   Exclude statements about:   - The presence or absence of supplies or materials in the Inner Setting, and instead code Inner Setting Domain: Available Resources. - Sharing marketing or branding innovation materials to engage individuals in implementation and/or delivery of the innovation, and instead code Implementation Process Domain: Engaging. - Actual provision of training, and instead code Inner Setting Domain: Access to Knowledge and Information. | Statements of the different components of the Safe PHM intervention: information, education, guideline, risk assessment, work equipment, practical education. |
| H. Innovation Cost | The innovation purchase and operating costs are affordable. | ***Note:*** *This construct captures the cost of the innovation, not the resources available in the Inner Setting to implement and deliver the innovation.*  Include statements about:   - Tangible costs to purchase, subscribe, or use the innovation; include cost of training associated with the innovation, e.g., registration.   Exclude statements about:   - Resources available (e.g., funding) to implement and deliver the innovation, and instead code Inner Setting Domain: Available Resources. - Time available to implement and deliver the innovation, and instead code Individuals Domain: Role * Opportunity. - Costs related to conducting research (e.g., funding for research staff, participant incentives); these are outside the purview of CFIR; however, a construct can be added to capture these statements. - Cost-effectiveness, and instead code Innovation Domain: Innovation Evidence-Base, Innovation Relative Advantage or as an Innovation Outcome where appropriate. | Statements on the costs of the innovation, but not the resources available in the Inner setting. |

## Outer Setting Domain Coding Guidelines

| **II. OUTER SETTING DOMAIN** | ***Outer Setting:*** The setting in which the Inner Setting exists, e.g., hospital system, school district, state. There may be multiple Outer Settings and/or multiple levels within the Outer Setting (e.g., community, system, state).  ***Project Outer Setting(s):*** [Document the actual Outer Setting in the project, e.g., type, location, and the boundary between the Outer Setting and the Inner Setting.] | | ***Study specific*** |
| --- | --- | --- | --- |
| **CFIR Construct Name** | **Construct Definition** *The degree to which:* | **Coding Guidelines**  Although constructs in this domain may influence implementation and delivery of the innovation in the Inner Setting, they all originate in the Outer Setting. As a result, teams must define the Inner and Outer Setting in their project, as well as the boundary between the two settings, in order to code accurately.  Add constructs to capture additional Outer Setting characteristics not included in CFIR. | **Any statements regarding**  **Outer setting** |
| A. Critical Incidents | Large-scale and/or unanticipated events disrupt implementation and/or delivery of the innovation. | Include statements about:   - Large-scale and/or unanticipated events, e.g., war, terrorism, natural disasters, infectious disease outbreaks/epidemics, government administration changes.   Exclude statements about:   - None specified |  |
| B. Local Attitudes | Sociocultural values (e.g., shared responsibility in helping recipients) and beliefs (e.g., convictions about the worthiness of recipients) encourage the Outer Setting to support implementation and/or delivery of the innovation. | ***Note:*** *This construct was added to CFIR to address situations in which the Inner Setting requires explicit support, e.g., participation or funding, from the Outer Setting to implement and/or deliver the innovation. If support from the Outer Setting is not needed, it’s unlikely this construct is relevant.*  Include statements about:   - Values and beliefs that encourage or discourage the Outer Setting from providing support, e.g., participation or funding, to the Inner Setting to implement and/or deliver the innovation in the Inner Setting.   - Sociocultural values, e.g., individualism vs. collectivism, equitable vs. equal distribution of support.   - Sociocultural beliefs, e.g., biases, stigma, discrimination, and/or oppression resulting from e.g., racism, ableism.   Exclude statements about:   - None specified |  |
| C. Local Conditions | Economic, environmental, political, and/or technological conditions enable the Outer Setting to support implementation and/or delivery of the innovation. | ***Note:*** *This construct was added to CFIR to address situations in which the Inner Setting requires explicit support, e.g., participation, funding, from the Outer Setting to implement and/or deliver the innovation. If support from the Outer Setting is not needed, it’s unlikely this construct is relevant.*  Include statements about:   - Economic, environmental, political, and/or technological conditions that enable or hinder the Outer Setting from providing support, e.g., participation or funding, to the Inner Setting to implement and/or deliver the innovation in the Inner Setting.   Exclude statements about:   - None specified |  |
| D. Partnerships & Connections | The Inner Setting is networked with external entities, including referral networks, academic affiliations, and professional organization networks. | Include statements about:   - Information sharing and co-learning through formal or informal connections with individuals or groups outside the Inner Setting, e.g., attending networking and/or educational events, being a member of a professional organization.   Exclude statements about:   - Partnerships and connections established to support implementation, i.e., as part of the implementation strategy, e.g., a Learning Collaborative, a Community of Practice, and instead define as part of the Implementation Process or add a construct as appropriate. - Relationships, networks, and teams inside the Inner Setting, and instead code Inner Setting Domain: Relational Connections. - Communication and information sharing practices inside the Inner Setting, and instead code Inner Setting Domain: Communications. |  |
| E. Policies & Laws | Legislation, regulations, professional group guidelines and recommendations, or accreditation standards support implementation and/or delivery of the innovation. | Include statements about:   - Any kind of policy, regulation, guideline, or law that originates in the Outer Setting relevant to implementation and/or delivery of the innovation.   Exclude statements about:   - Any kind of policy, regulation, guideline, or law that originates in the Inner Setting relevant to implementation and/or delivery of the innovation. |  |
| F. Financing | Funding from external entities (e.g., grants, reimbursement) is available to implement and/or deliver the innovation. | Include statements about:   - The availability of funding from the Outer Setting.   Exclude statements about:   - The availability of funding in the Inner Setting, and instead code Inner Setting Domain: Available Resources: Funding.   - ***Note:*** *If the level of external funding influences the availability of funding in the Inner Setting, code both Outer Setting Domain: Financing and Inner Setting Domain: Available Resources: Funding.* |  |
| G. External Pressure | External pressures drive implementation and/or delivery of the innovation. | This construct can be used to code general information about External Pressures. More detailed information can be coded using the subconstructs below or by adding new subconstructs. |  |
| 1. Societal Pressure | Mass media campaigns, advocacy groups, or social movements or protests drive implementation and/or delivery of the innovation. | Include statements about:   - None specified   Exclude statements about:   - None specified |  |
| 2. Market Pressure | Competing with and/or imitating peer entities drives implementation and/or delivery of the innovation. | Include statements about:   - Acting to “keep up with the Joneses”, i.e., to close a gap compared to another competitor or influential entity (whether gap is actual or perceived). - Level of demand in the market, e.g., supply and demand.   Exclude statements about:   - None specified |  |
| 3. Performance-Measurement Pressure | Quality or benchmarking metrics or established service goals drive implementation and/or delivery of the innovation. | Include statements about:   - Performance measures originating from the Outer Setting.   Exclude statements about:   - Performance measures originating from the Inner Setting, and instead code Inner Setting Domain: Incentive Systems.   - ***Note:*** *If Inner Setting individuals (e.g., High- or Mid-Level Leaders, deliverers) embrace and “own” external performance measures in the Inner Setting, code both Outer Setting Domain: External Pressure: Performance-Measurement Pressure and Inner Setting Domain: Incentive Systems.* |  |

## Inner Setting Domain Coding Guidelines

| **III. INNER SETTING DOMAIN** | ***Inner Setting:*** The setting in which the innovation is implemented, e.g., hospital, school, city. There may be multiple Inner Settings and/or multiple levels within the Inner Setting, e.g., unit, classroom, team.  ***Project Inner Setting(s):*** [Document the actual Inner Setting in the project, e.g., type, location, and the boundary between the Outer Setting and the Inner Setting.] | | ***Study specific*** |
| --- | --- | --- | --- |
| **CFIR Construct Name** | **Construct Definition** *The degree to which:* | **Coding Guidelines**  Constructs in this domain originate in the Inner Setting. As a result, teams must define the Inner and Outer Setting in their project, as well as the boundary between the two settings, in order to code accurately.  Add constructs to capture additional Inner Setting characteristics not included in CFIR. |  |
|  | ***Note:*** *Constructs A – D exist in the Inner Setting regardless of implementation and/or delivery of the innovation, i.e., they are persistent general characteristics of the Inner Setting.* | |  |
| A. Structural Characteristics | Infrastructure components support functional performance of the Inner Setting. | This construct can be used to code general information about Structural Characteristics of the Inner Setting. More detailed information can be coded using the subconstructs below or by adding new subconstructs. | Statements regarding the  Inner setting, i.e the health  care unit where the safe  PHM intervention is  implemented |
| 1. Physical Infrastructure | Layout and configuration of space and other tangible material features support functional performance of the Inner Setting. | Include statements about:   - General physical infrastructure of the Inner Setting, e.g., building size, layout.   Exclude statements about:   - Availability of space to implement and/or deliver the innovation, and instead code Inner Setting Domain: Available Resources: Space.   - ***Note:*** *If the general layout and configuration of the Inner Setting influences the availability of space to implement and/or deliver the innovation, code both Inner Setting Domain: Structural Characteristics: Physical Infrastructure and Available Resources: Space.* | Statements on the physical  infrastructure, such as  patient rooms or other  facilities at the unit |
| 2. Information Technology Infrastructure | Technological systems for tele-communication, electronic documentation, and data storage, management, reporting, and analysis support functional performance of the Inner Setting. | Include statements about:   - General IT infrastructure of the Inner Setting, e.g., Electronic Health Record (EHR) Systems, data warehouses, and data visualization tools.   Exclude statements about:   - Availability of IT equipment to implement and/or deliver the innovation, and instead code Inner Setting Domain: Available Resources: Materials & Equipment. | Statements on IT  infrastructure |
| 3. Work Infrastructure | Organization of tasks and responsibilities within and between individuals and teams, and general staffing levels, support functional performance of the Inner Setting. | Include statements about:   - General work infrastructure in the Inner Setting, e.g., levels of bureaucracy, rules or restrictions, reporting structure (power structures), delegation of tasks, schedules/shifts, order of tasks, workload, and work tempo (rate, rhythm or pattern of activities, pace). - General staffing level in the Inner Setting, e.g., high ratio of managers to total employees, high staff turnover, understaffing.   Exclude statements about:   - Availability of time (e.g., dedicated time) to implement and/or deliver the innovation, and instead code Individuals Domain: Role * Opportunity.   - ***Note:*** *If the general staffing levels in the Inner Setting influences the availability of time to implement and/or deliver the innovation, code both Inner Setting Domain: Structural Characteristics: Work Infrastructure and Individuals Domain: Role * Opportunity.* | Statements on work  infrastructure such as staff  levels, routines, schedules |
| B. Relational Connections | There are high quality formal and informal relationships, networks, and teams within and across Inner Setting boundaries (e.g., structural, professional). | Include statements about:   - General relational connections in the Inner Setting, e.g., level of boundary spanning, teamness, and cohesion. - Social network analyses in the Inner Setting.   Exclude statements about:   - Relationships with individuals and/or entities in the Outer Setting, and instead code Outer Setting Domain: Partnerships & Connections. - Forming an implementation team, and instead code Implementation Process Domain: Teaming. - Building relationships to engage deliverers and recipients, and instead code to Implementation Process Domain: Engaging.   - ***Note:*** *If engaging is being influenced by existing relationships, code both Inner Setting Domain: Relational Connections and Implementation Process Domain: Engaging.* | Statements on relational  connections |
| C. Communications | There are high quality formal and informal information sharing practices within and across Inner Setting boundaries (e.g., structural, professional). | Include statements about:   - General information sharing practices, e.g., how information is shared and received.   Exclude statements about:   - Receiving or not receiving information needed to implement and/or deliver the innovation, and instead code Inner Setting Domain: Access to Knowledge & Information.   - ***Note:*** *If Access to Knowledge & Information is being influenced by existing communication structures and norms, code both Inner Setting Domain: Communications and Access to Knowledge & Information.* - Communicating to engage individuals in implementation and/or delivery of the innovation, and instead code Implementation Process Domain: Engaging.   - ***Note:*** *If engaging is being influenced by existing communication structures and norms, code both Inner Setting Domain: Communications and Implementation Process Domain: Engaging.* | Statements on how  information is shared |
| D. Culture | There are shared values, beliefs, and norms across the Inner Setting. | This construct can be used to code general information about the culture of the Inner Setting. More detailed information can be coded using the subconstructs below or by adding new subconstructs. | Statements on the culture  of the unit |
| 1. Human Equality-Centeredness | There are shared values, beliefs, and norms about the inherent equal worth and value of all human beings. | Include statements about:   - General culture in the Inner Setting related to diversity, equity, inclusion and belonging (or lack thereof) of those who are marginalized and excluded due to systems of oppression, including but not limited to racism, sexism, heterosexism, cissexism, classism, ableism, and sizeism. - Shared power and decision making (or lack thereof) in the Inner Setting.   Exclude statements about:   - None specified |  |
| 2. Recipient-Centeredness | There are shared values, beliefs, and norms around caring, supporting, and addressing the needs and welfare of recipients. | Include statements about:   - General culture of Recipient-Centeredness in the Inner Setting, e.g., the extent to which the Inner Setting centers Recipients in decision making and designing processes.   Exclude statements about:   - Assessing recipient needs as part of the implementation process, and instead code Implementation Process Domain: Assessing Needs. - Needs of recipients, and instead code Individuals Domain: Innovation Recipients * Need. |  |
| 3. Deliverer-Centeredness | There are shared values, beliefs, and norms around caring, supporting, and addressing the needs and welfare of deliverers. | Include statements about:   - General culture of Deliverer-Centeredness in the Inner Setting, e.g., the extent to which the Inner Setting centers deliverers in decision making and designing processes.   Exclude statements about:   - Assessing deliverer needs as part of the implementation process, and instead code Implementation Process Domain: Assessing Needs. - Needs of deliverers, and instead code Individuals Domain: Innovation Deliverers * Need. |  |
| 4. Learning-Centeredness | There are shared values, beliefs, and norms around psychological safety, continual improvement, and using data to inform practice. | Include statements about:   - General culture of Learning-Centeredness in the Inner Setting, e.g., the extent to which the Inner Setting is improvement focused, learners are viewed as active agents with their own knowledge and experience, and failure is allowed. - The extent to which the Inner Setting is data driven, i.e., learning health systems turn data into knowledge, knowledge into performance, performance to data.   Exclude statements about:   - Collecting and discussing quantitative and qualitative information about the success/effectiveness of implementation or the innovation, and instead code Implementation Process Domain: Reflecting & Evaluating.   - ***Note:*** *If implementation and/or innovation specific Reflecting and Evaluating is being influenced by a learning-centered culture in the Inner Setting, code both Inner Setting Domain: Culture: Learning-Centeredness and Implementation Process Domain: Reflecting & Evaluating.* |  |
|  | ***Note:*** *Constructs E – K are specific to the implementation and/or delivery of the innovation****.*** | |  |
| E. Tension for Change | The current situation is intolerable and needs to change. | Include statements about:   - Needing or not needing to implement and/or deliver the innovation.   - ***Note:*** *Tension for Change is often associated with other constructs, e.g., if unmet recipient needs drive the need to implement the innovation, code both Individuals Domain: Innovation Recipients * Need and Inner Setting Domain: Tension for Change; if unmet performance measures drive the need to implement the innovation, code both Outer Setting Domain: External Pressure: Performance-Measurement Pressure and Inner Setting Domain: Tension for Change.*   Exclude statements about:   - The innovation being better or worse than current practice or an alternative innovation, and instead code Innovation Domain: Innovation Relative Advantage. | Statements about the need  or not the need to  implement safe PHM  intervention. Statements  about the intervention being  better or worse than current  practice - code in  Innovation Relative  Advantage. |
| F. Compatibility | The innovation fits with workflows, systems, and processes. | Include statements about:   - The level of fit between existing work processes and infrastructure and the innovation.   - ***Note:*** *If a lack of compatibility with Inner Setting workflows, systems, and processes drives adaptation, code both Inner Setting Domain: Compatibility and Implementation Process Domain: Adapting.*   Exclude statements about:   - Compatibility of the innovation with Inner Setting mission, and instead code Inner Setting Domain: Mission Alignment. | Statements about how the  Safe PHM intervention fits  with workflows etc. |
| G. Relative Priority | Implementing and delivering the innovation is important compared to other initiatives. | Include statements about:   - Having to manage multiple priorities in the Inner Setting, including other initiatives happening at the same time.   - ***Note:*** *Relative Priority is often associated with other constructs. For example, if priority is influenced by staffing levels, code both Inner Setting Domain: Work Infrastructure and Inner Setting Domain: Relative Priority; if priority is influenced by resources available to implement, code both Inner Setting Domain: Available Resources and Inner Setting Domain: Relative Priority.*   Exclude statements about:   - The innovation being/not being a perceived as a priority for recipients compared to other issues, and instead code Individuals Domain: Innovation Recipients * Capability, Opportunity, or Motivation as appropriate. | Statements about managing  multiple priorities at the unit |
| H. Incentive Systems | Tangible and/or intangible incentives and rewards and/or disincentives and punishments support implementation and delivery of the innovation. | Include statements about:   - Performance reviews, evaluations, appraisals, or mandates to help ensure accountability and/or tangible or intangible rewards or punishments. - Tangible extrinsic rewards/punishments: Promotions/demotions, pay raise/pay decrease. - Intangible extrinsic rewards/punishments: Praise/criticism, recognition/lack of recognition.   Exclude statements about:   - Intrinsic incentives, and instead code Individuals Domain: Roles * Motivation. - Performance measures originating from the Outer Setting, and instead code Outer Setting: External Pressure: Performance-Measurement Pressure.   - ***Note:*** *If Inner Setting individuals (e.g., High- or Mid-Level Leaders, Innovation Deliverers) embrace and “own” external performance measures in the Inner Setting, code both Outer Setting Domain: External Pressure: Performance-Measurement Pressure and Inner Setting Domain: Incentive Systems.* | Statements on incentives |
| I. Mission Alignment | Implementing and delivering the innovation is in line with the overarching commitment, purpose, or goals in the Inner Setting. | Include statements about:   - Degree of alignment between the innovation and the overarching commitment, purpose, or goals in the Inner Setting.   Exclude statements about:   - None specified | Statements about mission  alignment |
| J. Available Resources | Resources are available to implement and deliver the innovation. | This construct can be used to code general information about Available Resources in the Inner Setting. More detailed information can be coded using the subconstructs below or by adding new subconstructs. | Statements about available  resources to implement safe  intervention |
| 1. Funding | Funding is available to implement and deliver the innovation. | Include statements about:   - The availability and accessibility of funding to implement and/ or deliver the innovation in the Inner Setting.   Exclude statements about:   - The availability of funding from sources in the Outer Setting, and instead code Outer Setting Domain: Financing.   - ***Note:*** *If the level of external funding influences the availability of funding in the Inner Setting, code both Outer Setting Domain: Financing and Inner Setting Domain: Available Resources: Funding.* - Availability of funding to conduct research (e.g., obtaining regulatory approvals); this is outside the purview of CFIR; however, a construct can be added to capture these statements. |  |
| 2. Space | Physical space is available to implement and deliver the innovation. | Include statements about:   - The availability and accessibility, including the configuration, location, and quality of space, needed to implement and/or deliver the innovation.   Exclude statements about:   - The general layout and configuration of the Inner Setting, and instead code Inner Setting Domain: Structural Characteristics: Physical Infrastructure.   - ***Note:*** *If the general layout and configuration of the Inner Setting influences the availability of space to implement and/or deliver the innovation, code both Inner Setting Domain: Structural Characteristics: Physical Infrastructure and Available Resources: Space.* - The availability of space to conduct research (e.g., sufficiently private space to consent research participants); this is outside the purview of CFIR; however, a construct can be added to capture these statements. |  |
| 3. Materials & Equipment | Supplies are available to implement and deliver the innovation. | Include statements about:   - The availability and accessibility of materials and equipment needed to implement and/or deliver the innovation.   Exclude statements about:   - The design and quality of innovation materials, and instead code Innovation Domain: Innovation Design. - Availability of materials and equipment to conduct research (e.g., use of monitoring devices specifically for research purposes); this is outside the purview of CFIR; however, a construct can be added to capture these statements. |  |
| K. Access to Knowledge & Information | Guidance and/or training is accessible to implement and deliver the innovation. | Include statements about:   - The availability, convenience, accessibility, and usefulness of training (e.g., online learning modules, live training, workshops, on-demand) and guidance (e.g., ongoing real-time help, access to experts, informational materials, FAQs).   Exclude statements about:   - The presence or absence of implementation materials such as training and guidance (e.g., implementation toolkit, implementation plan) bundled with the innovation, and instead code Innovation Domain: Innovation Design.   - ***Note:*** *If access to information about implementing and/or delivering the innovation is being influenced by the presence or absence of implementation materials, code both Innovation Domain: Innovation Design and Inner Setting Domain: Access to Knowledge & Information.* - Existing communication structures and norms unrelated to implementation and/or delivery, and instead code Inner Setting Domain: Communications.   - ***Note:*** *If access to information about implementing and/or delivering the innovation is being influenced by existing communication structures and norms, code both Inner Setting Domain: Communications and Access to Knowledge & Information.* - Sharing information to engage individuals in implementation and/or delivery of the innovation, and instead code Implementation Process Domain: Engaging.   - ***Note:*** *If engaging is being completed by providing access to information about implementing and/or delivering the innovation, code both Inner Setting Domain: Access to Knowledge & Information and Implementation Process Domain: Engaging.* | Statements about the  availability and usefulness  of training and education.  For an example, how they  make use of the education |

## Individuals Domain Coding Guidelines

| **IV. INDIVIDUALS DOMAIN** | ***Individuals:*** The roles and characteristics of individuals. ***Note:*** *Roles may be internal or external to the Inner Setting.* | | ***Study specific*** |
| --- | --- | --- | --- |
| **ROLES SUBDOMAIN** | ***Project Roles:*** [Document the roles applicable to the project and their location in the Inner or Outer Setting.]  ***Note:*** *See the Characteristics Subdomain below for additional questions.* | |  |
| **CFIR Construct Name** | **Construct Definition** | **Coding Guidelines**  It is recommended to code the Role construct first, i.e., capture all data relevant to an individual under the Role construct first, and then code Characteristic constructs as appropriate within each role.  Roles may be internal or external to the Inner Setting and are not mutually exclusive, e.g., an Implementation Lead may also be a Mid-Level Leader.  Add constructs to capture additional Roles of individuals not included in CFIR. |  |
| A. High-level Leaders | Individuals with a high level of authority, including key decision-makers, executive leaders, or directors. | Include statements about:   - Formal high-level leaders, i.e., individuals that are officially recognized and have a high-level of power based on hierarchy.   Exclude statements about:   - Formal mid-level leaders, i.e., individuals that are officially recognized and have a mid-level of power based on hierarchy, and instead code Individuals Domain: Mid-Level Leaders. - Informal leaders, i.e., individuals that are not officially recognized but have influence based on their reputation, and instead code Individuals Domain: Opinion Leaders. - Implementation leaders, i.e., individuals leading implementation of the innovation, and instead code Individuals Domain: Implementation Leads.   - ***Note:*** *If an implementation leader is also another kind of leader, code both roles. For example, if the individual leading implementation is also the head of a department in the Inner setting, code both Individuals Domain: Implementation Leads and Mid-Level Leaders.* | Statements on higher-level  Leaders |
| B. Mid-level Leaders | Individuals with a moderate level of authority, including leaders supervised by a high-level leader and who supervise others. | Include statements about:   - Formal mid-level leaders, i.e., individuals that are officially recognized and have a mid-level of power based on hierarchy.   Exclude statements about:   - Formal high-level leaders, i.e., individuals that are officially recognized and have a high-level of power based on hierarchy, and instead code Individuals Domain: High-Level Leaders. - Informal leaders, i.e., individuals that are not officially recognized but have influence based on their reputation, and instead code Individuals Domain: Opinion Leaders. - Implementation leaders, i.e., individuals leading implementation of the innovation, and instead code Individuals Domain: Implementation Leads.   - ***Note:*** *If an Implementation Lead is also another kind of leader, code both roles. For example, if the individual leading implementation is also the head of a department in the Inner setting, code both Individuals Domain: Implementation Leads and Mid-Level Leaders.* | General statements about  mid-level leaders, but not  in the role as implementation  leaders |
| C. Opinion Leaders | Individuals with informal influence on the attitudes and behaviors of others. | Include statements about:   - Informal leaders, i.e., individuals that are not officially recognized but have influence based on their reputation.   Exclude statements about:   - Formal high-level leaders, i.e., individuals that are officially recognized and have a high-level of power based on hierarchy, and instead code Individuals Domain: High-Level Leaders. - Formal mid-level leaders, i.e., individuals that are officially recognized and have a mid-level of power based on hierarchy, and instead code Individuals Domain: Mid-Level Leaders. - Implementation leaders, i.e., individuals leading implementation of the innovation, and instead code Individuals Domain: Implementation Leads.   - ***Note:*** *If an Implementation Lead is also another kind of leader, code both roles. For example, if the individual leading implementation is also the head of a department in the Inner setting, code both Individuals Domain: Implementation Leads and Mid-Level Leaders.* |  |
| D. Implementation Facilitators | Individuals with subject matter expertise who assist, coach, or support implementation. | Include statements about:   - Individuals with subject matter and/or implementation expertise that support the Implementation Lead and Team Members.   Exclude statements about:   - None specified | Statements about the  external facilitators,  their role, mission and  support |
| E. Implementation Leads | Individuals who lead efforts to implement the innovation. | Include statements about:   - Implementation leaders, i.e., individuals leading implementation of the innovation.   - ***Note:*** *If an Implementation Lead is also another kind of leader, code both roles. For example, if the individual leading implementation is also the head of a department in the Inner setting, code both Individuals Domain: Implementation Leads and Mid-Level Leaders.*   Exclude statements about:   - Formal high-level leaders, i.e., individuals that are officially recognized and have a high-level of power based on hierarchy, and instead code Individuals Domain: High-Level Leaders. - Formal mid-level leaders, i.e., individuals that are officially recognized and have a mid-level of power based on hierarchy, and instead code Individuals Domain: Mid-Level Leaders. - Informal leaders, i.e., individuals that are not officially recognized but have influence based on their reputation, and instead code Individuals Domain: Opinion Leaders. | Statements about the  managers and how they  lead and support (or not)  efforts to implement the  safe PHM intervention |
| F. Implementation Team Members | Individuals who collaborate with and support the Implementation Leads to implement the innovation, ideally including Innovation Deliverers and Recipients. | Include statements about:   - Individuals who collaborate with and support the Implementation Leads to implement the innovation, ideally including deliverers and recipients.   Exclude statements about:   - Forming an implementation team, and instead code Implementation Process Domain: Teaming. | Statements about the  Implementation team, such  as their role, activities,  and support |
| G. Other Implementation Support | Individuals who support the Implementation Leads and/or Implementation Team Members to implement the innovation. | Include statements about:   - None specified   Exclude statements about:   - None specified |  |
| H. Innovation Deliverers | Individuals who are directly or indirectly delivering the innovation. | Include statements about:   - Individuals directly or indirectly involved with delivering the innovation, e.g., staff providing a program, staff referring recipients to the program.   - ***Note:*** *If there are multiple types of Deliverers, it is helpful to create subconstructs.*   Exclude statements about:   - None specified |  |
| I. Innovation Recipients | Individuals who are directly or indirectly receiving the innovation. | Include statements about:   - Individuals directly or indirectly receiving the innovation, e.g., patients and/or caregivers, students and/or parents.   - ***Note:*** *If there are multiple types of recipients, it is helpful to create subconstructs.*   Exclude statements about:   - None specified |  |
| **CHARACTERISTICS SUBDOMAIN** | ***Project Role Characteristics:*** [Document the characteristics applicable to the roles in the project based on the COM-B system [12] or role-specific theories. For example, theories related to:   - Behavior change, e.g., the Theoretical Domains Framework [13], [14], the Theory of Planned Behavior [15] or the Social Ecological Theory [16] may provide constructs more relevant for Innovation Recipients and Innovation Deliverers. - Facilitation [17], [18] and project management [19], [20] may provide constructs more relevant for Implementation Facilitators and Implementation Leads. - Leadership [17], [18] may provide constructs more relevant for High- and Mid-Level Leaders.   These role-specific constructs may be mapped to the broader COM-B constructs; for example, all 14 domains of the Theoretical Domains Framework (TDF) map to the COM-B system [12].]  ***Note:*** *See the Roles Subdomain above for additional questions. Some of the constructs in this domain may be more or less relevant depending on the associated role, e.g., most teams will want to assess Need related to Innovation Recipients, but that characteristic may not be relevant for other roles.* | |  |
| **CFIR Construct Name** | **Construct Definition:**  *The degree to which:* | **Coding Guidelines**  Users are advised to code the Role construct first, i.e., capture all data relevant to an individual under the Role construct first, and then code Characteristic constructs as appropriate within each role.  Add constructs to capture additional Characteristics of individuals not included in CFIR. | **Study specific** |
| A. Need | The individual(s) has deficits related to survival, well-being, or personal fulfillment, which will be addressed by implementation and/or delivery of the innovation. | Include statements about:   - None specified   Exclude statements about:   - Collecting information about needs of individuals, e.g., Innovation Recipients, to guide implementation and/or delivery of the innovation, and instead code Implementation Process Domain: Assessing Needs. |  |
| B. Capability | The individual(s) has interpersonal competence, knowledge, and skills to fulfill Role. | Include statements about:   - Psychological and physical ability to fulfill role, e.g., knowledge, skills, decision-making ability, physical strength.   Exclude statements about:   - Characteristics that are conferred onto individuals by the Inner or Outer Setting, e.g., the time and authority granted to the Implementation Lead to fulfill their role, and instead code Individuals Domain: Implementation Lead * Opportunity. | Statements about the  external facilitators and  implementation teams’  characteristics regarding:  B. Capabilities,  C. Opportunities, and  D. Motivation |
| C. Opportunity | The individual(s) has availability, scope, and power to fulfill Role. | Include statements about:   - Characteristics that are conferred onto individuals by the Inner or Outer Setting, e.g., the time and authority granted to the Implementation Lead to fulfill their role.   Exclude statements about:   - General staffing levels in the Inner Setting, e.g., high staff turnover, understaffing, and instead code Inner Setting Domain: Structural Characteristics: Work Infrastructure.   - ***Note:*** *If the general staffing level in the Inner Setting* *influences the availability of time to implement and/or deliver the innovation, code both Inner Setting Domain: Structural Characteristics: Work Infrastructure and Individuals Domain: Role * Opportunity.* - Availability of time to conduct research; this is outside the purview of CFIR; however, a construct can be added to capture these statements. |  |
| D. Motivation | The individual(s) is committed to fulfilling Role. | Include statements about:   - Receptivity, desire, and/or dedication to fulfill role.   Exclude statements about:   - None specified |  |

## Implementation Process Domain Coding Guidelines

| **V. IMPLEMENTATION PROCESS DOMAIN** | ***Implementation Process:*** The activities and strategies used to implement the innovation.  ***Project Implementation Process:*** [Document the implementation process framework [21] and/or activities and strategies [8], [9] being used to implement the innovation. Distinguish the implementation process used to implement the innovation (activities that end after implementation is complete) from the innovation (the “thing” that continues when implementation is complete) [2], [7], [10].] | |  |
| --- | --- | --- | --- |
| **CFIR Construct Name** | **Construct Definition:** *The degree to which individuals:* | **Coding Guidelines**  Though the constructs listed in the Implementation Process domain are commonly understood as important for successful implementation, this domain is not prescriptive. The processes described within these constructs may occur in a non-linear sequence or in incremental cycles of change.  Add constructs to capture additional implementation processes or strategies not included in CFIR. |  |
| A. Teaming | Join together, intentionally coordinating and collaborating on interdependent tasks, to implement the innovation. | Include statements about:   - The process of forming an implementation team, e.g., team building activities used to form and maintain a team.   Exclude statements about:   - Existing teamness, boundary spanning, and/or cohesion in the Inner Setting not related to the implementation process, and instead code Inner Setting Domain: Relational Connections.   - ***Note:*** *If existing relationships, networks, and teams within and across the Inner Setting influence the process of forming an implementation team, code both Inner Setting Domain: Relational Connections and Implementation Process Domain: Teaming.* - The roles and characteristics of the team and individual team members, and instead code the appropriate constructs in the Individuals domain.   - ***Note:*** *If CFIR users want to code team characteristics, a Team role can be added to the Individuals Domain or Team can be added as a level in the Inner Setting. See Inner Setting note.* | Statements on how  implementation teams  were formed |
| B. Assessing Needs | Collect information about priorities, preferences, and needs of people. | This construct can be used to code general information about Assessing Needs as part of the Implementation Process. More detailed information can be coded using the subconstructs below or by adding new subconstructs. |  |
| 1. Innovation Deliverers | Collect information about the priorities, preferences, and needs of deliverers to guide implementation and delivery of the innovation. | Include statements about:   - The process of assessing deliverer needs, e.g., completing informal or formal interviews or surveys, to guide implementation and delivery of the innovation.   Exclude statements about:   - The characteristics and/or priorities, preferences, and needs of Innovation Deliverers, and instead code Individuals Domain: Innovation Deliverers * Characteristics. |  |
| 2. Innovation Recipients | Collect information about the priorities, preferences, and needs of recipients to guide implementation and delivery of the innovation. | Include statements about:   - The process of assessing recipient needs, e.g., completing informal or formal interviews or surveys, to guide implementation and delivery of the innovation.   Exclude statements about:   - The characteristics and/or priorities, preferences, and needs of Innovation Recipients, and instead code Individuals Domain: Innovation Recipients * Characteristics. |  |
| C. Assessing Context | Collect information to identify and appraise barriers and facilitators to implementation and delivery of the innovation. | Include statements about:   - The process of assessing context, e.g., using CFIR to conduct informal or formal interviews or surveys, to guide implementation and delivery of the innovation 😉   Exclude statements about:   - None specified |  |
| D. Planning | Identify roles and responsibilities, outline specific steps and milestones, and define goals and measures for implementation success in advance. | Include statements about:   - The process of making and/or refining an implementation plan, e.g., identifying roles and responsibilities, setting goals.   Exclude statements about:   - The existence of a written implementation plan provided with the innovation, and instead code Innovation Domain: Innovation Design. - Monitoring or reflecting on progress towards implementation goals, and instead code Implementation Process Domain: Reflecting & Evaluating: Implementation. |  |
| E. Tailoring Strategies | Choose and operationalize implementation strategies to address barriers, leverage facilitators, and fit context. | Include statements about:   - The process of choosing and operationalizing strategies, e.g., selecting strategies to address known barriers to implementation.   - ***Note:*** *If assessing context guides selection of strategies, code both Implementation Process Domain: Assessing Context and Tailoring Strategies.*   Exclude statements about:   - Adaptations to the innovation, and instead code Implementation Process Domain: Adapting. |  |
| F. Engaging | Attract and encourage participation in implementation and/or the innovation. | This construct can be used to code general information about Engaging as part of the Implementation Process. More detailed information can be coded using the subconstructs below or by adding new subconstructs. | Statements on how the  implementation team,  facilitator and/or the leader  engaged participation |
| 1. Innovation Deliverers | Attract and encourage deliverers to serve on the implementation team and/or to deliver the innovation. | Include statements about:   - The process of engaging and retaining deliverers, e.g., sharing/disseminating information or evidence about the innovation and branding or marketing materials to “sell” the innovation or mandating or “voluntelling” involvement.   Exclude statements about:   - The degree of delivery or enactment of expected behaviors by Innovation Deliverers, and instead code Individuals Domain: Innovation Deliverers * Motivation or Actual Implementation Outcomes as appropriate. |  |
| 2. Innovation Recipients | Attract and encourage recipients to serve on the implementation team and/or participate in the innovation. | Include statements about:   - The process of engaging and retaining recipients, e.g., sharing information or evidence about the innovation and branding or marketing materials to “sell” the innovation or mandating or “voluntelling” involvement.   Exclude statements about:   - The degree of receipt or enactment of expected behaviors by Innovation Recipients, and instead code Individuals Domain: Innovation Recipients * Motivation or Innovation Outcomes: Recipient Impacts as appropriate. |  |
| G. Doing | Implement in small steps, tests, or cycles of change to trial and cumulatively optimize delivery of the innovation. | ***Note:*** *This construct captures the process of trialing the innovation,* ***not*** *the need to trial nor the inherent trialability of the innovation.*  Include statements about:   - The process of implementing the innovation, e.g., completing Plan-Do-Study-Act cycles.   Exclude statements about:   - The (in)ability to trial the innovation, due to features of the innovation itself, and instead code Innovation Domain: Innovation Trialability. | Statements on how the  implementation team,  facilitator and/or leader  implemented the safe  PHM intervention  (What work did they do  and how) |
| H. Reflecting & Evaluating | Collect and discuss quantitative and qualitative information about the success of implementation and the innovation. | This construct can be used to code general information about Reflecting & Evaluating as part of the Implementation Process. More detailed information can be coded using the subconstructs below or by adding new subconstructs. | Statements on reflection and  Evaluation of the  Implementation and  Intervention |
| 1. Implementation | Collect and discuss quantitative and qualitive information about the success of implementation. | Include statements about:   - The process of reflecting and evaluating on the progress and/or success of implementation, e.g., time spent reviewing, discussing, and/or analyzing (i.e., making sense of) information related to the progress and/or success of implementation.   Exclude statements about:   - The actual progress and/or success of implementation, and instead code Actual Implementation Outcomes [11]. - The extent to which the Inner Setting has shared values around continual improvement and using data to inform practice, and instead code Inner Setting Domain: Culture: Learning-Centeredness Culture.   - ***Note:*** *If implementation and/or innovation specific Reflecting and Evaluating is being influenced by a learning-centered culture in the Inner Setting, code both Inner Setting Domain: Culture: Learning-Centeredness and Implementation Process Domain: Reflecting & Evaluating.* - Identifying barriers and facilitators to implementation of the innovation, and instead code Implementation Process Domain: Assessing Context. - Setting implementation goals, and instead code Implementation Process Domain: Planning. - An individual’s reflections as a part of data collection (e.g., participating in an interview), and instead code the appropriate construct based on the content of the reflection. |  |
| 2. Innovation | Collect and discuss quantitative and qualitative information about the success of the innovation. | Include statements about:   - The process of reflecting and evaluating on the progress and/or success of the innovation, e.g., time spent reviewing, discussing, and/or analyzing (i.e., making sense of) information related to the progress and/or success of the innovation.   Exclude statements about:   - The actual progress and/or success of the innovation, and instead code Innovation Outcomes [11]. - The extent to which the Inner Setting has shared values around continual improvement and using data to inform practice, and instead code Inner Setting Domain: Culture: Learning-Centeredness Culture.   - ***Note:*** *If implementation and/or innovation specific Reflecting and Evaluating is being influenced by a learning-centered culture in the Inner Setting, code both Inner Setting Domain: Culture: Learning-Centeredness and Implementation Process Domain: Reflecting & Evaluating.* - Pre-existing evidence supporting innovation effectiveness, and instead code Innovation Domain: Innovation Evidence-Base. - An individual’s reflections as a part of data collection (e.g., participating in an interview), and instead code the appropriate construct based on the content of the reflection. |  |
| I. Adapting | Modify the innovation and/or the Inner Setting for optimal fit and integration into work processes. | ***Note:*** *This construct captures the process of adapting the innovation and/or Inner Setting,* ***not*** *the need to adapt nor the inherent adaptability of the innovation. Users may wish to add new subconstructs based on a reporting framework for adaptations, e.g., the* [*FRAME*](https://pubmed.ncbi.nlm.nih.gov/31171014/)*.*  Include statements about:   - The process of adapting the innovation, e.g., changing innovation components or processes, or the Inner Setting, e.g., changing Inner Setting work processes.   - ***Note:*** *If adaptations are driven by other CFIR constructs, code both constructs. For example:*     - *If recipient capability, opportunity, motivation, or need drive adaptations, code both Individuals Domain: Innovation Recipients * Characteristic and Implementation Process Domain: Adapting.*     - *If deliverer capability, opportunity, motivation, or need drive adaptations, code both Individuals Domains: Innovation Deliverers * Characteristics and Implementation Process Domain: Adapting.*     - *If a lack of compatibility with Inner Setting workflows, systems, and processes drives adaptation, code both Inner Setting Domain: Compatibility and Implementation Process Domain: Adapting.*   Exclude statements about:   - The (in)ability to adapt the innovation, due to features of the innovation itself, e.g., a rigid protocol or lack of “permission” to change components, and instead code Innovation Domain: Innovation Adaptability. - The innovation needing/not needing to be adapted, and instead code the relevant CFIR construct, e.g., if an innovation needs/needed to be adapted because it did not fit with existing workflows, code Inner Setting Domain: Compatibility. - Adapting implementation strategies and/or implementation processes, and instead code the relevant strategy or process being adapted, e.g., changes to the implementation plan would be coded to Implementation Process Domain: Planning. | Statements on modification  on the intervention or  the setting (unit) for  optimal fit |

## Outcomes Addendum Coding Guidelines

| **OUTCOMES ADDENDUM** | ***Note:*** *While outcomes are not a CFIR domain, it is often important to collect and analyze data related to implementation outcomes, which in turn lead to innovation outcomes.* | |  |
| --- | --- | --- | --- |
| **Outcome Name** | **Outcome Definition:** | **Coding Guidelines** | **Study specific** |
| Implementation Outcomes | Outcomes that capture the success or failure of implementation. | |  |
| Anticipated Implementation Outcomes | Predictions of future implementation success or failure, i.e., implementation outcomes that have not yet occurred.  ***Note:*** *These outcomes are forward-looking; constellations of CFIR determinants across domains predict these outcomes.* | | Not applicable |
| Adoptability | The likelihood key decision-makers will decide to put the innovation in place/innovation deliverers will decide to deliver to innovation. | Include statements about:   - The predicted complexity and/or difficulty of adopting the innovation. - Progress towards adoption goals.   Exclude statements about:   - None specified |  |
| Implementability | The likelihood the innovation will be put in place or delivered. | Include statements about:   - The predicted complexity and/or difficulty of implementing the innovation. - Progress toward implementation goals.   Exclude statements about:   - None specified |  |
| Sustainability | The likelihood the innovation will be put in place or delivered over the long-term. | Include statements about:   - The predicted complexity and/or difficulty of sustaining the innovation. - Progress towards sustainment goals.   Exclude statements about:   - None specified |  |
| Actual Implementation Outcomes | Observed (current or past) implementation success or failure, i.e., implementation outcomes that have occurred.  ***Note:*** *These outcomes are backward-looking; constellations of CFIR determinants across domains explain these outcomes* | |  |
| Adoption | The extent key decision-makers decide to put the innovation in place/innovation deliverers decide to deliver the innovation. | Include statements about:   - None specified   Exclude statements about:   - Outcomes that capture the success or failure of the innovation, and instead code Innovation Outcomes. | Statements about key decisions  to deliver the safe PHM  intervention |
| Implementation | The extent the innovation is in place or being delivered. | Include statements about:   - None specified   Exclude statements about:   - Outcomes that capture the success or failure of the innovation, and instead code Innovation Outcomes. | Statements about the extent  the safe PHM intervention is  integrated into ordinary practice,  and how |
| Sustainment | The extent the innovation is in place or being delivered over the long-term. | Include statements about:   - None specified   Exclude statements about:   - Outcomes that capture the success or failure of the innovation, and instead code Innovation Outcomes. | Statements about the extent  the safe PHM intervention is  being delivered over the long-  term |
| Innovation Outcomes | Outcomes capturing the success or failure of the innovation, based on the impact of the innovation on three important constituents: innovation recipients, innovation deliverers, and key decision-makers. | | Not applicable |
| Innovation Effectiveness | The extent to which the Innovation is effective. | Include statements about:   - Actual innovation outcomes in the Inner Setting e.g., in a retrospective evaluation, a statement such as “The innovation was effective for our patients."   Exclude statements about:   - Outcomes that capture the success or failure of implementation, and instead code Implementation Outcomes. - Anticipated innovation outcomes in the Inner Setting, e.g., “We think the innovation will be effective for our patients” and code Innovation Evidence-Base. |  |
| Key Decision-Maker Impacts | The effect or influence the innovation has on key decision-makers and/or the system. | Include statements about:   - Types of impact, including positive and negative impacts, e.g., unintended consequences of the innovation, on key decision-makers.   Exclude statements about:   - None specified |  |
| Deliverer Impacts | The effect or influence the innovation has on deliverers. | Include statements about:   - Types of impact, including positive and negative impacts, e.g., unintended consequences of the innovation, on deliverers.   Exclude statements about:   - None specified |  |
| Recipient Impacts | The effect or influence the innovation has on recipients. | Include statements about:   - Recipient reach (or create new construct). - Types of impact, including positive and negative impacts, e.g., unintended consequences of the innovation, on recipients.   Exclude statements about:   - None specified |  |

# References

[1]	Saldana, J., *The coding manual for qualitative researchers*, 2nd ed. SAGE, 2015.

[2]	G. M. Curran, “Implementation science made too simple: a teaching tool.,” *Implement Sci Commun*, vol. 1, p. 27, 2020, doi: 10.1186/s43058-020-00001-z.

[3]	L. Albrecht, M. Archibald, D. Arseneau, and S. D. Scott, “Development of a checklist to assess the quality of reporting of knowledge translation interventions using the Workgroup for Intervention Development and Evaluation Research (WIDER) recommendations,” *Implementation Sci*, vol. 8, no. 1, p. 52, Dec. 2013, doi: 10.1186/1748-5908-8-52.

[4]	M. Butler *et al.*, “AHRQ series on complex intervention systematic reviews—paper 3: adapting frameworks to develop protocols,” *Journal of Clinical Epidemiology*, vol. 90, pp. 19–27, Oct. 2017, doi: 10.1016/j.jclinepi.2017.06.013.

[5]	The AIMD Writing/Working Group, P. Bragge, J. M. Grimshaw, C. Lokker, and H. Colquhoun, “AIMD - a validated, simplified framework of interventions to promote and integrate evidence into health practices, systems, and policies,” *BMC Med Res Methodol*, vol. 17, no. 1, p. 38, Dec. 2017, doi: 10.1186/s12874-017-0314-8.

[6]	T. C. Hoffmann *et al.*, “Better reporting of interventions: template for intervention description and replication (TIDieR) checklist and guide,” *BMJ*, vol. 348, p. g1687, Mar. 2014, doi: 10.1136/bmj.g1687.

[7]	R. Lengnick-Hall *et al.*, “Six practical recommendations for improved implementation outcomes reporting,” *Implementation Sci*, vol. 17, no. 1, p. 16, Dec. 2022, doi: 10.1186/s13012-021-01183-3.

[8]	B. J. Powell *et al.*, “A compilation of strategies for implementing clinical innovations in health and mental health,” *Med Care Res Rev*, vol. 69, no. 2, pp. 123–57, Apr. 2012, doi: 10.1177/1077558711430690.

[9]	B. J. Powell *et al.*, “A refined compilation of implementation strategies: results from the Expert Recommendations for Implementing Change (ERIC) project,” *Implementation Science*, vol. 10, no. 1, p. 21, 2015.

[10]	H. Pinnock *et al.*, “Standards for Reporting Implementation Studies (StaRI) Statement,” *BMJ*, p. i6795, Mar. 2017, doi: 10.1136/bmj.i6795.

[11]	L. J. Damschroder, C. M. Reardon, M. A. Opra Widerquist, and J. Lowery, “Conceptualizing outcomes for use with the Consolidated Framework for Implementation Research (CFIR): the CFIR Outcomes Addendum,” *Implementation Sci*, vol. 17, no. 1, p. 7, Dec. 2022, doi: 10.1186/s13012-021-01181-5.

[12]	S. Michie, M. M. van Stralen, and R. West, “The behaviour change wheel: A new method for characterising and designing behaviour change interventions,” *Implement Sci*, vol. 6, p. 42, Apr. 2011, doi: 10.1186/1748-5908-6-42.

[13]	S. Michie *et al.*, “Making psychological theory useful for implementing evidence based practice: a consensus approach,” *Qual Saf Health Care*, vol. 14, no. 1, pp. 26–33, Feb. 2005, doi: 10.1136/qshc.2004.011155.

[14]	J. Cane, D. O’Connor, and S. Michie, “Validation of the theoretical domains framework for use in behaviour change and implementation research,” *Implementation Sci*, vol. 7, no. 1, p. 37, Dec. 2012, doi: 10.1186/1748-5908-7-37.

[15]	I. Ajzen, “The theory of planned behaviour: Reactions and reflections,” *Psychology & Health*, vol. 26, no. 9, pp. 1113–1127, Sep. 2011, doi: 10.1080/08870446.2011.613995.

[16]	D. Stokols, “Translating Social Ecological Theory into Guidelines for Community Health Promotion,” *Am J Health Promot*, vol. 10, no. 4, pp. 282–298, Mar. 1996, doi: 10.4278/0890-1171-10.4.282.

[17]	A. Metz, L. Louison, K. Burke, and C. Ward, “Implementation Support Practitioner Profile,” National Implementation Research Network, 2020. Accessed: Dec. 22, 2021. [Online]. Available: https://nirn.fpg.unc.edu/resources/implementation-support-practitioner-profile

[18]	B. Albers, A. Metz, and K. Burke, “Implementation support practitioners – a proposal for consolidating a diverse evidence base,” *BMC Health Serv Res*, vol. 20, no. 1, p. 368, Dec. 2020, doi: 10.1186/s12913-020-05145-1.

[19]	M. Barron and A. Barron, “Project Management Areas of Expertise,” in *Project Management*. [Online]. Available: https://cnx.org/contents/XpF315mY@11.6:_nDfs3nk@2/Project-Management-Areas-of-Expertise

[20]	R. Müller and R. Turner, “Leadership competency profiles of successful project managers,” *International Journal of Project Management*, vol. 28, no. 5, pp. 437–448, Jul. 2010, doi: 10.1016/j.ijproman.2009.09.003.

[21]	P. Nilsen, “Making sense of implementation theories, models and frameworks,” *Implementation science*, vol. 10, no. 1, Art. no. 1, 2015.
