# Supplementary material for: Stakeholders’ experiences of the implementation of a multifactorial intervention for safe patient handling and movement: a qualitative explorative and descriptive study
Source: BMC Health Serv Res. 2026 Nov 6;26:1545. doi: 10.1186/s12913-026-16068-8 (PMC13741535; doi:10.1186/s12913-026-16068-8)
Supplement: Supplementary file 3 [file 12913_2026_16068_MOESM3_ESM.docx]

**Reflexive statement from the main analyst (ENS)**

I am a registered physiotherapist with over 15 years of experience of clinical work, mostly in primary care but also at hospitals and within elderly care in Sweden, Western Europe. I started my working life as an assistant nurse in elderly homes and at a hospital. As a teacher at a university, I have provided student nurses with education in safe patient handling and movement (PHM). As such, I have empirical familiarity with PHM which has shaped my understanding of PHM as a multifaceted practice involving the patient as well as different professions, cultures, and regulations.

I have been part of the broader research programme on patient and worker safety since its initiation, but I did not participate in carrying out the cluster randomised controlled trial (RCT). My preconception, based on empirical and scientific knowledge, is that safe PHM practices are crucial in healthcare, but this can only be achieved by a theoretically informed and structured implementation of an intervention that is based on engaged leadership, a participatory approach, and realistic plans for sustainment.

I am an experienced qualitative researcher, mostly familiar with the research methodology of qualitative content analysis. My epistemological and ontological point of view is constructivism, where I see reality as multiple and subjective, truth as relative and changeable, and results/knowledge as being created in interactions between the interviewer/researcher and the informants.

When CW and I planned the interviews, we were careful not to interview any informant whom we had met before. The reason for this was to avoid power imbalances and to ensure an open atmosphere where the informants could share positive and negative experiences. We thoroughly discussed within the research group who should analyse the data and how our preconceptions should be handled during the analysis process. We decided that I should be the main analyst, being an experienced qualitative researcher who was not mainly responsible for or actively involved in the RCT.

I recognised that knowledge is not passively received in qualitative research, but instead actively co-constructed between the researcher and the informant. However, I approached the data analysis with awareness that my previous knowledge of PHM could colour my reading of the data. When coding the data, I carefully coded the manifest content, close to the text. The codes were then mapped into a theoretical framework. The consistency of preliminary categories (internal homogenous and external heterogeneous) and the descriptions of the categories’ content were discussed within the research group, where JS and GS had a more outside perspective. In that way, my and CW’s preconceptions could be held back and the informants’ descriptions emphasised. The regular discussions were pivotal in challenging my assumptions and expanding my analytic perspective. They also ensured that the emerging categories were not merely a projection of my own worldview, but the result of a careful and critically grounded engagement with the data.
