# Supplementary material for: Stakeholders’ experiences of the implementation of a multifactorial intervention for safe patient handling and movement: a qualitative explorative and descriptive study
Source: BMC Health Serv Res. 2026 Nov 6;26:1545. doi: 10.1186/s12913-026-16068-8 (PMC13741535; doi:10.1186/s12913-026-16068-8)
Supplement: Supplementary file 4 [file 12913_2026_16068_MOESM4_ESM.docx]

**Figure 1**. The Implementation Research Logic Model, based on the model presentation of Smith, Li, & Rafferty 2020 and Moore et al. 2011 (1, 2), illustrates the proposed relationships between determinants, strategies, intervention, mechanisms and outcomes in this research project. The logic model was developed during the preparation phase and was complemented with findings from this qualitative study. Implementation strategies were based on the Expert Recommendation for Implementing Change (ERIC) taxonomy (3) with an emphasis on facilitation, as described in i-PARIHS (4). The logic model includes determinants in the outer setting, inner setting, characteristics of individuals, intervention characteristics and process, based the Consolidated Framework for Implementation Research (CFIR) (5). Determinants, mechanisms of impact of the strategies and outcomes were developed through the analysis of the stakeholder interviews. Outcomes refer to effectiveness of the multifactorial safe patient handling and movement intervention (PHM) and of implementation of the intervention. * Reported elsewhere; # not measured in this project

**Figure 2.** Overview of the process evaluation of the entire research project, including the process evaluation’s components, research questions, and data sources. In this manuscript, only data collected from interviews are presented. Figure adapted from Saarijärvi et al. 2020 (6).

**References**

1.	Moore GF, Audrey S, Barker M, Bond L, Bonell C, Hardeman W, et al. Process evaluation of complex interventions: Medical Research Council guidance. Bmj. 2015;350:h1258.

2.	Smith JD, Li DH, Rafferty MR. The Implementation Research Logic Model: a method for planning, executing, reporting, and synthesizing implementation projects. Implement Sci. 2020;15(1):84.

3.	Powell BJ, Waltz TJ, Chinman MJ, Damschroder LJ, Smith JL, Matthieu MM, et al. A refined compilation of implementation strategies: results from the Expert Recommendations for Implementing Change (ERIC) project. Implement Sci. 2015;10:21.

4.	Harvey G, Kitson A. PARIHS revisited: from heuristic to integrated framework for the successful implementation of knowledge into practice. Implement Sci. 2016;11:33.

5.	Damschroder LJ, Reardon CM, Widerquist MAO, Lowery J. The updated Consolidated Framework for Implementation Research based on user feedback. Implement Sci. 2022;17(1):75.
